# Maternal Avocado Consumption and Pregnancy and Newborn Outcomes: Prospective Kuopio Birth Cohort

**DOI:** 10.1111/jog.70561

**Published:** 2026-11-05

**Authors:** Feon W. Cheng, Nikki A. Ford, Katri Backman, Raimo Voutilainen, Markku Pasanen, Leea Keski‐Nisula, Soili M. Lehto, Sari Hantunen

**Affiliations:** ^1^ Avocado Nutrition Center, Hass Avocado Board Mission Viejo California USA; ^2^ Institute of Clinical Medicine, School of Medicine University of Eastern Finland Kuopio Finland; ^3^ Department of Pediatrics Kuopio University Hospital Kuopio Finland; ^4^ School of Pharmacy University of Eastern Finland Kuopio Finland; ^5^ Department of Obstetrics and Gynecology Kuopio University Hospital Kuopio Finland; ^6^ Institute of Clinical Medicine University of Oslo Oslo Norway; ^7^ R&D Department, Division of Mental Health Services Akershus University Hospital Lørenskog Norway; ^8^ Department of Psychiatry University of Helsinki and Helsinki University Hospital Helsinki Finland; ^9^ Institute of Public Health and Clinical Nutrition University of Eastern Finland Kuopio Finland

**Keywords:** avocado, maternal diet, newborn outcomes, pregnancy outcomes, prenatal nutrition

## Abstract

**Background:**

Maternal fruit and vegetable intake during pregnancy has been associated with improved pregnancy and newborn outcomes. However, the role of individual foods—specifically avocados, which are rich in key micronutrients—has not been studied in relation to perinatal health. Thus, this study aimed to examine expectant mother avocado consumption and its association with pregnancy and newborn outcomes using the Kuopio Birth Cohort (KuBiCo)—a prospective cohort study.

**Methods:**

Maternal avocado intake was assessed using a validated food frequency questionnaire in the first and third trimesters. Avocado consumers were defined as participants who reported consuming any avocado (> 0 g) during pregnancy, and avocado non‐consumers were defined as those who didn't report consuming any avocado (0 g). Pregnancy outcomes included preeclampsia, gestational hypertension, gestational diabetes, excessive bleeding during childbirth, and delivery by cesarean section. Early neonatal outcomes included being large or small for gestational age, low birth weight, macrosomia, preterm delivery, admission to neonatal intensive care, diagnosis of developmental disorder, and Apgar scores at 1 and 5 min. Logistic regression models were used to estimate associations between maternal avocado consumption and pregnancy and neonatal outcomes, adjusting for relevant covariates.

**Results:**

Pregnant individuals who consumed avocados (mean intake: 19.5 g/day) had 26.5% lower odds of undergoing a cesarean delivery compared to those who did not, even when adjusting for relevant covariates (e.g., overall diet quality). No statistically significant associations were observed for other pregnancy and newborn outcomes.

**Conclusion:**

Avocado consumption during pregnancy was associated with lower odds of cesarean delivery, even when accounting for potential confounding covariates.

## 1 Introduction

Consuming fruits, vegetables, and a generally healthy diet during pregnancy has been associated with improved pregnancy and newborn outcomes, including reduced risk of gestational diabetes, hypertensive disorders, and adverse birth outcomes [1, 2, 3, 4]. These benefits are attributed to vitamins, phytochemicals, and dietary fiber, which can support glucose regulation, improve insulin sensitivity, enhance endothelial function, and reduce oxidative stress [5, 6, 7, 8, 9].

While understanding the overall benefits of healthy eating patterns, including fruit and vegetable consumption, is valuable, examining individual foods may provide more practical and actionable insights. For example, Sun et al. found that different fruits were associated with varying risks of gestational diabetes, likely due to differences in nutrient composition [4]. This level of specificity is valuable for clinical counseling, where pregnant individuals may inquire about specific foods.

Avocados are nutrient‐dense and provide a variety of bioactive compounds, including monounsaturated fats, fiber, potassium, magnesium, and folate—all of which may support metabolic and cardiovascular health [10]. Prior studies have linked avocado consumption to improved glucose control, lipid profiles, and body weight regulation in non‐pregnant populations [11, 12, 13, 14, 15]. Given that maternal metabolic health and vascular function are important predictors of pregnancy outcomes, such as preeclampsia and gestational hypertension, avocado intake during pregnancy may be beneficial [16, 17, 18]. Evidence from prior studies [1, 2] suggests that adherence to healthy dietary patterns is associated with lower odds of hypertensive disorders of pregnancy, supporting a plausible role for nutrient‐dense foods in influencing these outcomes. Additionally, findings from non‐pregnant women indicate that avocado consumption is associated with a lower incidence of hypertension [19]. Emerging research further highlights the potential importance of avocado intake during pregnancy. For example, a recent study found that maternal avocado consumption during pregnancy was associated with lower odds of infant food allergy at 12 months, even after adjusting for relevant covariates [20]. Collectively, these findings suggest that avocado intake may affect both maternal and infant health outcomes.

Therefore, this study aimed to investigate the association between expectant maternal avocado consumption and pregnancy and newborn outcomes using data from the Kuopio Birth Cohort (KuBiCo). Hypertensive disorders of pregnancy (preeclampsia and gestational hypertension) were considered primary outcomes based on prior evidence, while other pregnancy and newborn outcomes were examined as secondary (exploratory) outcomes. The hypothesis was that expectant mothers who consume avocados during pregnancy have improved pregnancy and newborn outcomes compared to those who do not consume avocados.

## 2 Methods

### 2.1 Study Population

KuBiCo, launched in 2012, is a prospective cohort study aiming to enroll up to 10 000 mother‐infant pairs [21]. The primary goal of KuBiCo is to explore how genetic, environmental, and lifestyle factors during gestation affect maternal and child health [22]. Pregnant women planning to give birth at Kuopio University Hospital (KUH) in Finland were invited to join KuBiCo, with over 90% enrolled during the first trimester at their initial prenatal visits.

Between 2013 and 2022, 7944 women participated in KuBiCo, representing 37.7% of all KUH births during this period. Participants were representative of Finnish mothers in terms of maternal demographics, gestational age at delivery, neonatal sex, and birth weight [22]. Data collection included clinical evaluations, standardized questionnaires, and biological samples throughout pregnancy and at birth. Longitudinal follow‐up of mothers and children continues annually through surveys and clinical assessments. Additionally, KuBiCo data are integrated with the hospital's birth register and subsequently linked to national health registries to support long‐term epidemiological research [22]. More comprehensive information about the KuBiCo study has been detailed in earlier publications [22, 23, 24, 25].

### 2.2 Dietary Assessment

Dietary data collection commenced in March 2013. This analysis includes participants who completed nutrition‐specific questionnaires between March 2013 and November 2022. Dietary intake was assessed during the first and third trimesters using a web‐based Food Frequency Questionnaire (FFQ), modified from the Kuopio Breast Cancer Study instrument [26]. The KuBiCo FFQ includes 163 items with standardized portion sizes, and participants reported their habitual intake frequency over the preceding 3 months for each item (e.g., avocado), using a nine‐category response scale. Daily intakes (g/day) were computed by multiplying the reported intake frequency by portion size. Nutrient and energy intakes were estimated using the 2023 version of the Finland's National Food Composition Database (Fineli) [27].

Eligible KuBiCo participants were dichotomized into avocado consumers and non‐consumers. For analyses of pregnancy outcomes, avocado consumers were defined as individuals reporting any avocado intake (> 0 g) during the first trimester, while non‐consumers reported no intake (0 g) during that period. For analyses of newborn outcomes, consumers included those who reported avocado intake (> 0 g) in either the first or third trimester, while non‐consumers reported no intake (0 g) across both trimesters.

### 2.3 Pregnancy Outcomes

Pregnancy outcomes included preeclampsia, gestational hypertension, gestational diabetes (GDM), significant bleeding during childbirth (defined as > 1000 mL), and delivery by cesarean section. All outcomes were categorized as binary variables (yes or no).

Pregnant women and parturients were diagnosed by attending physicians with various pregnancy and labor complications either during their hospital stay or at prenatal visits to outpatient maternity clinics. These complications were documented in the KUH birth records using specific ICD‐10 codes. Preclampsia and gestational hypertension were identified with using ICD‐10 codes O11, O13, O14, O15, and O16.

Gestational diabetes (GDM) was diagnosed in accordance with Finnish national guidelines [28]. In brief, most pregnant women underwent a 75‐g oral glucose tolerance test (OGTT) following an overnight fast, typically between 24 and 28 weeks of gestation. Women classified as high‐risk—such as those with polycystic ovary syndrome, a history of GDM or earlier delivery of a macrosomic infant, glucosuria in early pregnancy, use of oral corticosteroids, body mass index (BMI) > 35 kg/m^2^, or a family history of Type 2 diabetes—were screened earlier, between 12 and 16 weeks of gestation, and retested at 24–28 weeks if initial results were normal. However, OGTT screening is not universally applied. According to the Finnish national guidelines, testing is not required for nulliparous women aged < 25 years with a BMI between 18.5 and 24.9 kg/m^2^ and no family history of Type 2 diabetes, or for primiparous or multiparous women aged < 40 years with the same BMI range and no history of GDM or fetal macrosomia in previous pregnancies. Consequently, approximately 70%–80% of pregnant women in Finland undergo OGTT during pregnancy [28]. GDM was defined based on plasma glucose thresholds of fasting ≥ 5.3 mmol/L, 1‐h ≥ 10.0 mmol/L, or 2‐h ≥ 8.6 mmol/L. Diagnoses were identified in the KUH birth records using ICD‐10 O24.4 or O24.9 codes, as documented in hospital and birth registry data.

### 2.4 Newborn Outcomes

Newborn outcomes included large for gestational age (LGA) (birth weight > 90th percentile), LGA (birth weight > 2 standard deviation), small for gestational age (SGA) (birth weight < 10th percentile), SGA (birth weight < 2 standard deviation), low birth weight (birth weight < 2500 g), macrosomia (birth weight > 4500 g), preterm delivery (< 37 gestational weeks), admission to neonatal intensive care (NICU), diagnosis of developmental disorder after birth (ICD‐10 Q Codes), 1‐min Apgar score (< 7), and 5‐min Apgar score (< 7) [29, 30, 31]. Outcomes were categorized as binary variables.

### 2.5 Covariates

The following maternal covariates were included: maternal age at delivery (years, continuous), marital status (categorized as married, cohabitation, other relationship, divorced or widow, or single), educational attainment (16 years or less or more than 16 years), parity (nulliparous/primiparous or multiparous), body mass index (BMI kg/m^2^) in the first trimester (continuous), gestational age at delivery (weeks, continuous), alcohol intake as a percentage of total daily energy intake during the first and third trimesters (continuous), Edinburgh Postnatal Depression Scale (EPDS) score during the first trimester (continuous), diet quality in the first and third trimesters (continuous), and self‐reported smoking status (categorized as non‐smoker, quit during first trimester, active smoker, unknown, smoked before pregnancy, quit after first trimester, or passive smoker).

During the first trimester, KuBiCo participants completed the EPDS via a web‐based questionnaire. The EPDS is a validated screening tool for postpartum depression, consisting of 10 items rated on a 4‐point Likert scale, yielding a total score ranging from 0 to 30 [32].

The Alternative Healthy Eating Index for Pregnancy (AHEI‐P) assesses diet quality using data from FFQ administered during the first and third trimesters. Adapted from the original Alternative Healthy Eating Index (AHEI), a validated tool for evaluating diet quality in relation to chronic disease risk [33], the AHEI‐P was modified to reflect the unique nutritional needs of pregnancy. These modifications include the inclusion of folate, calcium, and iron—key nutrients during pregnancy—and the exclusion of the alcohol component [34]. Due to potential allergen concerns, the nuts and soy protein components were removed; instead, tofu and soybeans were incorporated into the vegetable component. The AHEI‐P comprises nine components: vegetables, fruits, the ratio of white to red meat, fiber, trans fats, the ratio of polyunsaturated to saturated fatty acids, folate, calcium, and iron. Each component is scored from 0 to 10 based on adherence to pregnancy‐specific dietary recommendations, yielding a total score ranging from 0 to 90. Higher scores indicate better overall diet quality.

### 2.6 Statistical Analyses

Descriptive statistics, chi‐square tests, and independent sample *t*‐tests compared characteristics between avocado consumers and non‐consumers during pregnancy. Logistic regression models assessed associations between the avocado consumer group and pregnancy and newborn outcomes. Model 1 was unadjusted for both pregnancy and newborn outcomes. For pregnancy outcomes analyses, Model 2 was adjusted for maternal age, marital status, education, nulliparity, BMI (Trimester 1—T1), alcohol consumption (T1), EPDS (T1), diet quality (AHEI‐P) (T1), and smoking. For newborn outcomes analyses, Model 2 was adjusted for maternal age, marital status, education, nulliparity, body mass index (T1), gestational age at delivery, alcohol consumption (T3), EPDS (T1), diet quality (AHEI‐P) (T3), smoking, gestational diabetes, preeclampsia, and gestational hypertension. Gestational age at delivery was not adjusted for preterm delivery. 1‐min Apgar score (< 7) and 5‐min Apgar score (< 7) were further adjusted for cesarean section because of their association documented in the literature [35, 36].

Avocado intake in this cohort was highly skewed; nevertheless, potential dose–response relationships were explored. Avocado intake was categorized into three groups: no intake (“never”), moderate intake (> 0–8 g/day), and higher intake (> 8 g/day). These tertile‐derived categories resulted in unequal group sizes because of the highly skewed distribution and the presence of tied values. For example, in the pregnancy outcome analysis, the no‐intake group included 1136 participants, the moderate‐intake group included 536 participants, and the higher‐intake group included 323 participants. In addition, avocado intake was modeled as a continuous variable to further assess dose–response associations.

To evaluate the potential impact of healthy‐user bias and residual confounding, several sensitivity analyses were performed. First, stepwise adjustment models were constructed by adding covariates in conceptually grouped blocks to examine how the association between avocado intake and outcomes of interest changed with the inclusion of additional confounders. The models were specified as follows: Model 1, unadjusted (avocado only); Model 2, additionally adjusted for maternal age and parity (baseline reproductive factors); Model 3, further adjusted for education and marital status (socioeconomic characteristics); Model 4, additionally adjusted for early pregnancy BMI (anthropometric factor); and Model 5, further adjusted for smoking, alcohol intake, EPDS, and AHEI‐P. Second, inverse probability of treatment weighting (IPTW) was applied. A propensity score for avocado intake was estimated using the same covariates included in the fully adjusted model. Stabilized weights were calculated, trimmed at the 1st and 99th percentiles to limit the influence of extreme values, and incorporated into a weighted logistic regression model with avocado intake as the exposure. Finally, the E‐value was calculated for the fully adjusted estimate to quantify the minimum strength of association that an unmeasured confounder would need to have with both the exposure and the outcome (on the risk ratio scale), beyond the variables already accounted for, to fully explain the observed association. This metric helps assess the plausibility that residual, unmeasured confounding could account for the findings.

Multicollinearity was assessed but not observed (i.e., tolerance > 0.2 and variance inflation factor ≤ 2.5). IBM SPSS Statistics 29, Armonk, NY was used to conduct analyses. *p*‐values < 0.05 were considered significant.

### 2.7 Protocol Registration and Checklist

The study protocol was developed and registered on the Open Science Framework (OSF) at https://osf.io/m864f. Additionally, the study adhered to the Strengthening the Reporting of Observational Studies in Epidemiology (STROBE) guidelines to ensure high‐quality reporting.

## 3 Results

### 3.1 Pregnancy Outcomes Analysis

Of the 5402 participants, 1995 were eligible for the pregnancy outcomes analysis (Figure 1a). Among them, 859 (43.1%) reported consuming avocado during pregnancy (Table 1). Avocado consumers were generally older (mean age 31.6 vs. 30.9 years, *p* = 0.004), had higher education levels (61.9% vs. 51.4% with > 16 years of education, *p* < 0.001), and were more often nulli‐ or primiparous (87.0% vs. 80.0%, *p* < 0.001). They also had significantly lower BMI in the first trimester (24.7 vs. 25.8 kg/m^2^, *p* < 0.001) and better diet quality as indicated by AHEI‐P scores (61.0 vs. 55.1, *p* < 0.001). Avocado consumers were also less likely to be smokers (*p* < 0.001) and to deliver by cesarean section (*p* = 0.020).

**FIGURE 1:**
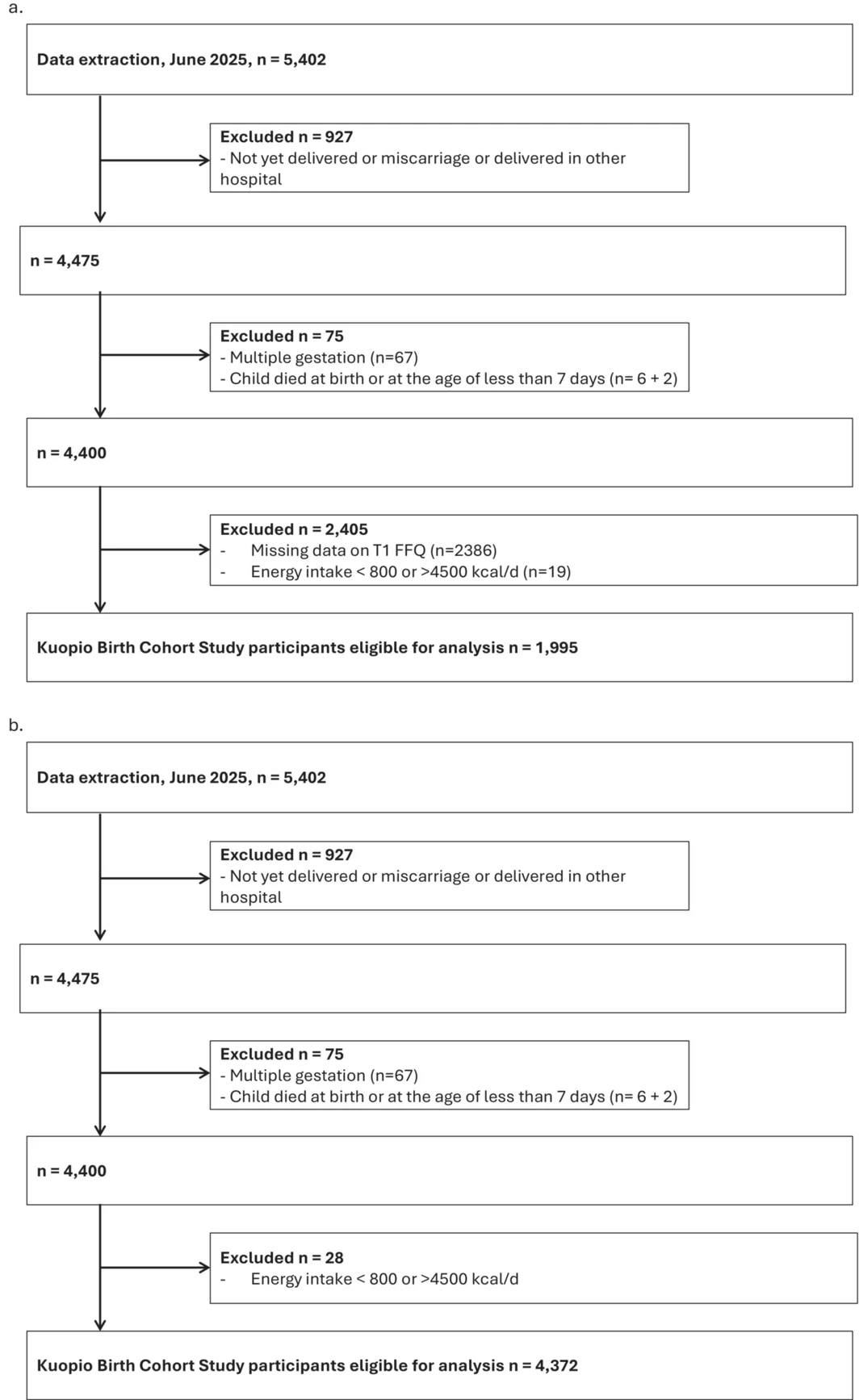
Flow chart of eligible Kuopio Birth Cohort Study (KuBiCo) participants included in (a) pregnancy‐related outcomes analyses and (b) newborn‐related outcomes analyses.

**TABLE 1:** Maternal characteristics of avocado consumers and non‐consumers in the pregnancy outcomes (*n* = 1995) and newborn outcomes analyses (*n* = 4372) of the Kuopio Birth Cohort Study, Finland^a^.

Maternal characteristics	Pregnancy outcome analysis				Newborn outcomes analysis			
	All (*n* = 1995)	Avocado non‐consumer during pregnancy (*n* = 1136)	Avocado consumer during pregnancy (*n* = 859)^b^	*p*	All (*n* = 4372)	Avocado non‐consumer during pregnancy (*n* = 2352)	Avocado consumer during pregnancy (*n* = 2020)^c^	*p*
Avocado consumption (g/d)	8.4 (19.8)	0.0	19.5 (26.4)	< 0.001	8.1 (18.0)	0.0	17.5 (23.2)	< 0.001
Maternal age at delivery (year)	31.2 (4.7)	30.9 (4.9)	31.6 (4.4)	0.004	31.1 (4.9)	30.7 (5.2)	31.6 (4.5)	< 0.001
Marital status, %				0.215				0.138
Married	54.9	54.2	55.9		53.5	52.3	54.9	
Cohabitation	38.2	38.0	38.5		39.0	39.3	38.7	
Other relationship	3.6	4.2	2.8		3.8	4.2	3.3	
Divorced or widow	3.2	3.6	2.8		3.6	4.0	3.0	
Single	0.1	0.1	0.1		0.2	0.2	0.1	
Education, %				< 0.001				0.004
16 years or less	44.0	48.6	38.1		40.0	42.0	37.7	
More than 16 years	56.0	51.4	61.9		60.0	58.0	62.3	
Parity, %				< 0.001				< 0.001
Nulli‐ or primiparous	83.0	80.0	87.0		81.4	77.8	85.6	
Multiparous	17.0	20.0	13.0		18.6	22.2	14.4	
Body mass index at the first trimester (kg/m^2^)	25.3 (5.2)	25.8 (5.5)	24.7 (4.7)	< 0.001	25.2 (5.1)	25.7 (5.5)	24.5 (4.6)	< 0.001
Gestational age at delivery (weeks)	39.3 (1.6)	39.2 (1.6)	39.3 (1.6)	0.396	39.3 (1.6)	39.3 (1.6)	39.4 (1.6)	0.549
Cesarean delivery, %	13.3	15.0	11.3	0.026	11.9	12.7	10.2	< 0.001
Admitted to neonatal intensive care unit, %	11.8	12.9	10.2	0.066	11.0	11.7	10.1	0.146
Alcohol consumption (percentage of total energy/day)	0.30 (0.77)	0.30 (0.80)	0.29 (0.74)	0.930	0.01 (0.08)	0.01 (0.09)	0.01 (0.06)	0.432
EPDS scores	4.6 (4.2)	4.7 (4.4)	4.5 (3.9)	0.467	4.7 (4.1)	4.8 (4.2)	4.6 (3.9)	0.542
Diet quality (AHEI‐P)	57.6 (10.9)	55.1 (11.1)	61.0 (9.6)	< 0.001	58.6 (10.3)	55.3 (10.9)	61.0 (9.5)	< 0.001
Smoking during pregnancy, %				< 0.001				< 0.001
Not smoking	81.3	77.0	87.6		80.2	75.2	86.0	
Stopping smoking at 1st trimester	9.2	11.4	6.2		10.0	12.4	7.2	
Smoking	1.8	2.7	1.0		1.7	2.6	0.7	
Not known	0.7	0.8	0.5		0.8	1.1	0.5	
Smoking before pregnancy	3.3	4.1	2.3		3.5	4.3	2.6	
Stopped smoking after 1st trimester	0.3	0.3	0.2		0.9	1.3	0.4	
Passive smoking	3.0	3.6	2.2		2.8	3.1	2.4	

Abbreviations: AHEI‐P, Alternative Healthy Eating Index for Pregnancy; BMI, body mass index; EPDS, Edinburgh Postnatal Depression Scale.

^a^ Means (standard deviation) for continuous variables and percentages for categorical variables.

^b^ Avocado consumers were identified as Kuopio Birth Cohort Study participants who reported consuming any amount of avocado in their food frequency questionnaire at Trimester 1.

^c^ Avocado consumers were identified as Kuopio Birth Cohort Study participants who reported consuming any amount of avocado in their food frequency questionnaire at trimester 1 and/or trimester 3.

The rate of cesarean delivery was significantly higher among non‐consumers (15%) when compared to consumers (11.3%) (*p* = 0.046 in model 2) (Table 2 and Figure 2a). Compared to avocado non‐consumers (during the first trimester), avocado consumers had 26.5% lower odds of cesarean delivery, even when adjusted for relevant covariates (Table 2). Sensitivity analyses yielded consistent results across stepwise models. The IPTW analysis produced a similar estimate (OR = 0.721, 95% CI 0.541–0.962; *p* = 0.026). The *E*‐value (~1.92) suggests that an unmeasured confounder would need to be associated with both avocado intake and cesarean delivery by nearly a twofold risk ratio, beyond the measured covariates, to fully explain the observed association—an effect size larger than typically observed in perinatal nutrition research.

**TABLE 2:** Odds ratio (OR) and 95% confidence intervals (CI) of reported pregnancy outcomes comparing between avocado consumers and non‐consumers during pregnancy (*n* = 1995)^a^.

Pregnancy outcomes	Model^b^	*N*	Odds ratio	95% CI	*p*
Preeclampsia	1	1948	0.907	0.534–1.542	0.719
	2	1948	1.061	0.605–1.863	0.835
Gestational hypertension	1	1948	0.671	0.387–1.161	0.151
	2	1948	0.755	0.420–1.356	0.347
Gestational diabetes	1	1948	0.939	0.757–1.166	0.571
	2	1948	1.131	0.888–1.440	0.320
Bleeding during childbirth	1	1813	0.984	0.698–1.387	0.927
	2	1813	0.893	0.623–1.279	0.536
Cesarean section	1	1740	0.724	0.545–0.962	0.026
	2	1740	0.735	0.543–0.995	0.046

Abbreviations: AHEI‐P, Alternative Healthy Eating Index for Pregnancy; BMI, body mass index; EPDS, Edinburgh Postnatal Depression Scale; T, trimester.

^a^ Reference = avocado non‐consumer.

^b^ Model 1: Unadjusted; Model 2: Adjusted for maternal age, marital status, education, nulliparity, BMI (T1), alcohol consumption (T1), EPDS, diet quality (AHEI‐P) (T1), and smoking.

**FIGURE 2:**
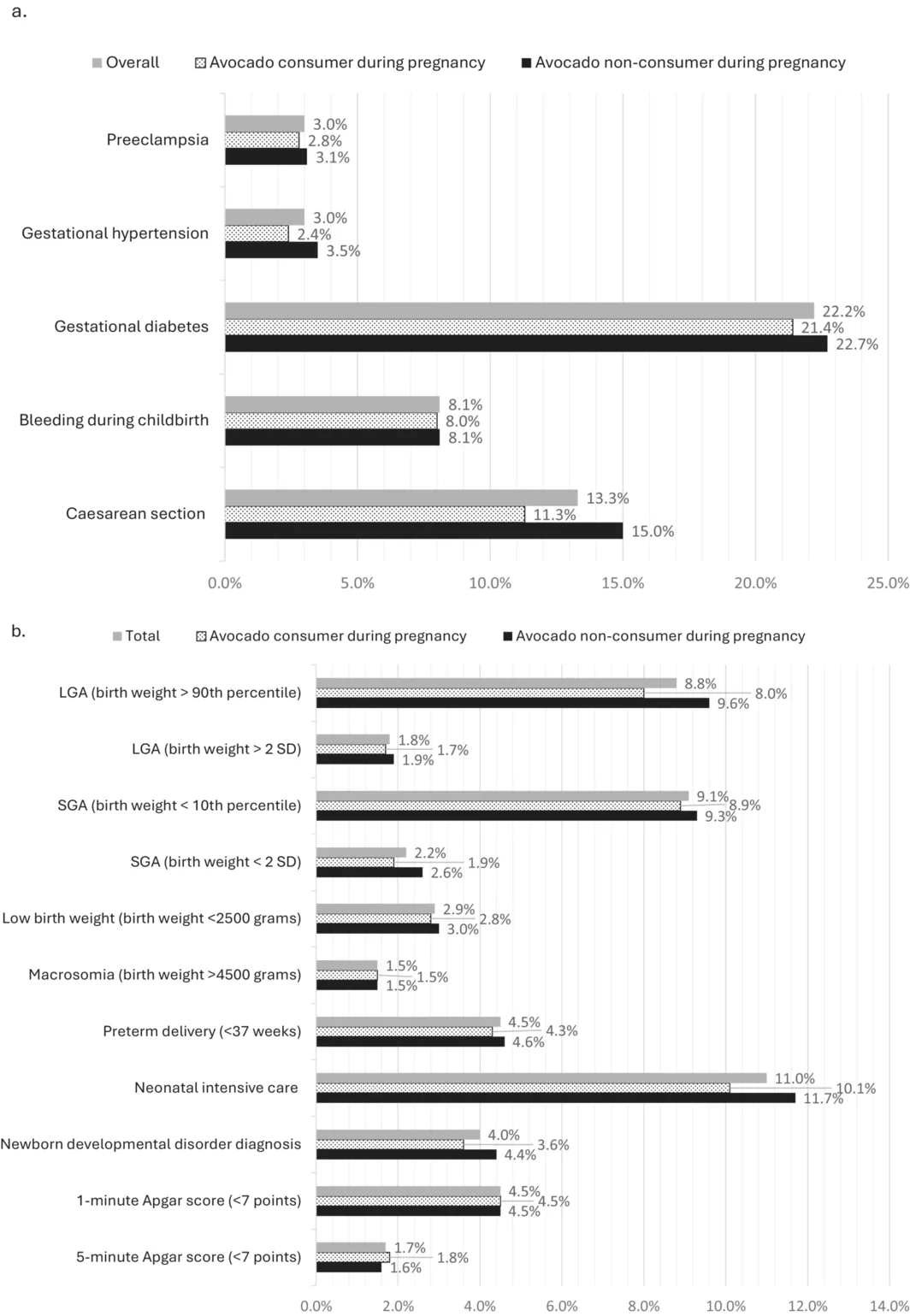
(a) Percentage of pregnancy‐related events by avocado consumer vs. non‐consumer from the eligible Kuopio Birth Cohort (KuBiCo) Study participants (*n* = 1995). Bleeding during childbirth is defined as bleeding over 1000 mL during childbirth. The bar fill patterns distinguish between avocado consumers and non‐consumers during pregnancy. A dotted pattern represents consumers, a solid black fill indicates non‐consumers, and a solid gray fill indicates all. (b) Percentage of newborn‐related events by avocado consumer vs. non‐consumer from the eligible Kuopio Birth Cohort (KuBiCo) Study participants (*n* = 4372). LGA, large for gestational age. SD, standard deviation. SGA, small for gestational age. The bar fill patterns distinguish between avocado consumers and non‐consumers during pregnancy. A dotted pattern represents consumers, a solid black fill indicates non‐consumers, and a solid gray fill indicates all.

No associations were observed for the development of preeclampsia, gestational hypertension, gestational diabetes, or excessive bleeding during childbirth after adjusting for maternal and lifestyle factors.

In dose–response analyses, avocado intake was categorized into three levels (0 g, > 0–8 g, and > 8 g/day). Moderate intake (> 0–8 g/day) was associated with lower odds of cesarean delivery (OR 0.624; 95% CI 0.434–0.896), whereas higher intake (> 8 g/day) was not significantly associated (OR 0.945; 95% CI 0.632–1.412). When avocado intake was modeled as a continuous variable, no significant association with cesarean delivery was observed (OR 0.999; 95% CI 0.991–1.007; *p* = 0.795). No dose–response associations were observed for other pregnancy outcomes (*p* > 0.05).

### 3.2 Newborn Outcomes Analysis

Of the 5402 participants, 4372 were included in the newborn outcomes analysis (Figure 2b). Of these, 2020 (46.2%) women reported avocado consumption during pregnancy (Table 1). Similar to the pregnancy outcomes sample, avocado consumers were more likely to be older (mean age 31.6 vs. 30.7 years, *p* < 0.001), had higher education attainment (*p* = 0.004), and were more likely to be nulli‐ or primiparous (*p* < 0.001). They also had better diet quality (mean AHEI‐P score 61.0 vs. 55.3, *p* < 0.001) and smoked less during pregnancy (86.0% vs. 75.2%, *p* < 0.001). The birth by cesarean section was less common among avocado consumers (10.2% vs. 12.7%, *p* < 0.001). Other maternal or birth characteristics, such as marital status, BMI, gestational age at delivery, and NICU admission, did not differ significantly between groups.

Table 3 summarizes associations between avocado intake during pregnancy and various newborn outcomes. No statistically significant associations were observed in the adjusted model (Model 2) for any outcome assessed, including LGA (based on either of the two definitions), SGA (based on either of the two definitions), low birth weight, macrosomia, preterm birth, NICU admission, diagnosis of a newborn developmental disorder, or Apgar scores. A trend toward reduced odds of NICU admission was observed among neonates of avocado‐consuming mothers (10.1%) compared to those of non‐consuming mothers (11.7%) in the unadjusted model (*p* = 0.084), although the association was not statistically significant in the fully adjusted model (*p* = 0.195) (Table 3 and Figure 2b). When avocado intake was analyzed across three intake groups (0 g, > 0–8 g, > 8 g/day) for dose–response, no significant associations were observed for any newborn outcomes (*p* > 0.05), consistent with the binary intake analyses.

**TABLE 3:** Odds ratio (OR) and 95% confidence intervals (CI) of newborn outcomes comparing avocado consumers and non‐consumers during pregnancy in the Kuopio Birth Cohort (KuBiCo) study (*n* = 4372)^a^.

Newborn outcomes	Model^b^	*N*	Odds ratio	95% CI	*p*
LGA (birth weight > 90th percentile)	1	4372	0.819	0.663–1.012	0.064
	2	4372	0.890	0.708–1.119	0.317
LGA (birth weight > 2 SD)	1	4372	0.898	0.572–1.411	0.641
	2	4372	0.974	0.599–1.582	0.915
SGA (birth weight < 10th percentile)	1	4372	0.952	0.774–1.171	0.640
	2	4372	0.869	0.695–1.086	0.217
SGA (birth weight < 2 SD)	1	4372	0.732	0.486–1.104	0.137
	2	4372	0.827	0.531–1.288	0.401
Low birth weight (birth weight < 2500 g)	1	4372	0.933	0.655–1.329	0.700
	2	4372	1.502	0.851–2.649	0.160
Macrosomia (birth weight > 4500 g)	1	4372	1.003	0.618–1.627	0.991
	2	4372	1.150	0.683–1.939	0.599
Preterm delivery (< 37 weeks)^**c**^	1	4372	0.915	0.685–1.222	0.547
	2	4372	0.922	0.672–1.265	0.616
Neonatal intensive care	1	4371	0.845	0.697–1.023	0.084
	2	4371	0.866	0.697–1.077	0.195
Newborn developmental disorder diagnosis	1	4372	0.819	0.603–1.112	0.200
	2	4372	0.859	0.619–1.192	0.363
1‐min Apgar score (< 7 points)^d^	1	4372	1.000	0.750–1.331	0.998
	2	4372	0.975	0.716–1.328	0.875
5‐min Apgar score (< 7 points)^d^	1	4372	1.135	0.715–1.803	0.591
	2	4372	1.077	0.653–1.777	0.771

Abbreviations: AHEI‐P, Alternative Healthy Eating Index for Pregnancy; BMI, body mass index; EPDS, Edinburgh Postnatal Depression Scale; LGA, large for gestational age; SD, standard deviation; SGA, small for gestational age; T, trimester.

^a^ Reference = avocado non‐consumer.

^b^ Model 1: Unadjusted; Model 2: Adjusted for maternal age, marital status, education, nulliparity, BMI (T1), gestational age at delivery, alcohol consumption (T3), EPDS, diet quality (AHEI‐P) (T3), smoking, gestational diabetes, preeclampsia, and gestational hypertension.

^c^ Preterm delivery was not adjusted gestational age at delivery in Model 2.

^d^ Apgar scores were also adjusted for cesarean section in Model 2.

## 4 Discussion

This prospective cohort study investigated the relationship between maternal avocado consumption during pregnancy and a range of pregnancy and newborn outcomes using data from the KuBiCo Study. The findings suggest that avocado consumption during pregnancy is associated with a reduced likelihood of cesarean section delivery, even after adjusting for multiple potential confounders, such as the overall diet quality. However, no statistically significant associations were observed between avocado consumption and other pregnancy complications, including preeclampsia, gestational hypertension, gestational diabetes, or excessive bleeding during childbirth. Similarly, maternal avocado consumption was not significantly associated with any of the newborn outcomes, such as birthweight categories, gestational age at birth, NICU admission, or developmental disorders.

The prevalence of pregnancy‐ and newborn outcomes observed in this study is generally comparable to prior reported estimates, though some differences were observed. The proportion of women with GDM was slightly higher than the 2019 Finnish national estimate (22.2% vs. 19.1%) [23]. Based on data from the nationwide Medical Birth Register of women covering deliveries between 2004 and 2014, the rates of gestational hypertension and preeclampsia were 2.3% and 1.6%, respectively [37], whereas this study identified a slightly higher rate of 3% for both conditions. The cesarean section rate in the current sample (13.3%) was collected between 2013 and 2023. The proportion of cesarean sections increased over time and now exceeds 20%. A similar trend has been observed nationally, with rates increasing from 16.7% in 2014 to 20.3% in 2024 [38].

For newborn outcomes, SGA and LGA were classified using both percentile‐ [39] and SD‐based [24, 31] criteria. As expected, stricter SD‐based definitions resulted in lower estimates: 2.2% for SGA and 1.8% for LGA, compared to percentile‐based estimates (9.1% and 8.8%, respectively) [40]. The prevalence of SGA observed in this study was comparable to that reported in a Norwegian cohort [41] and a Finnish birth cohort [42]. The LGA prevalence of 8.8% was slightly lower than the 11.9% reported in earlier studies [40]. In comparison with 2023 national statistics from the Finnish Institute for Health and Welfare, this study reported slightly lower prevalences of low birth weight (2.9% vs. 4.1%), macrosomia (1.5% vs. 2.2%), NICU admissions (11% vs. 13.5%), and preterm birth (4.5% vs. 5.6%) [38]. The proportion of infants with a 5‐min Apgar score below 7 was 1.7%, consistent with prior findings [43].

This study is among the first to examine the association between avocado consumption and mode of delivery. While direct research on this topic is limited, previous studies have explored the relationship between overall dietary patterns and mode of delivery. In line with our findings, a meta‐analysis of five studies reported a trend toward lower odds of cesarean delivery among women adhering to healthy dietary plans [1]. Avocados align well with such diets, as they are nutrient‐dense and provide a wide array of essential vitamins, minerals, and phytochemicals [10, 44]. Several biological mechanisms may plausibly contribute to the observed association, although these remain speculative given the relatively low average intake in this cohort (19.5 g/day). First, half of an avocado contains approximately 6.7 g of monounsaturated fats, 4.6 g of dietary fiber, 345 mg of potassium, 19.5 mg of magnesium, and 60 μg of folate—nutrients known to support maternal metabolic and vascular health [45, 46]. For instance, monounsaturated fats and magnesium may contribute to improved vascular function and uterine muscle tone, which could theoretically influence labor progression and reduce the likelihood of surgical delivery [47, 48]. These avocado‐derived nutrients may affect maternal nutritional and metabolic status and, in turn, influence clinical pathways relevant to mode of delivery. Failure to progress in labor and fetal distress are two leading indications for cesarean delivery in Finland [49]. Maternal nutritional and metabolic status may influence myometrial function and uterine contractility, with implications for labor progression, while maternal vascular and placental function may influence fetal tolerance of labor [45, 50, 51, 52]. However, these pathways were not directly evaluated in the present study, and whether the intake levels observed were sufficient to induce meaningful physiological effects remains uncertain. Second, excessive gestational weight gain is a risk factor for cesarean delivery [53]. While no studies have specifically examined avocado consumption during pregnancy and gestational weight gain, dietary fiber and balanced fat intake have been linked to improved maternal weight trajectories and metabolic health, which may in turn support a favorable delivery mode [54, 55]. Moreover, evidence from non‐pregnant populations suggests that avocado consumption is associated with improved diet quality, reduced body weight, and increased satiety measures [56, 57, 58], further supporting its potential role in healthy weight management during pregnancy. Third, avocados contain bioactive compounds—such as lutein, vitamin E, and other antioxidants—that may help reduce systemic inflammation and oxidative stress [10, 52, 59]. Elevated inflammatory and oxidative markers have been linked to adverse pregnancy outcomes [60, 61, 62, 63]—suggesting a potential biological pathway through which diet may influence delivery mode. This is particularly relevant in this population, where most cesarean sections are performed because of medical indications rather than by maternal request [49]. However, the small number of cesarean deliveries by type limited the ability to examine the association according to cesarean section type. Future studies should consider higher intake levels, repeated dietary measures, and mechanistic biomarkers to better clarify potential pathways.

No significant associations were observed between avocado consumption during pregnancy and other pregnancy outcomes, including preeclampsia, gestational hypertension, GDM, or excessive bleeding during childbirth. While no previous studies have specifically examined avocado intake in relation to these outcomes, research has assessed the impact of broader dietary patterns, food group, and specific nutrients—such as monounsaturated fats—on these conditions, but the results are mixed. For example, Radesky et al. reported no significant association between monounsaturated fat intake during pregnancy and the risk of impaired glucose tolerance or GDM [64]. Similarly, a meta‐analysis of 17 studies found no significant association between adherence to a healthy dietary pattern and GDM risk [1]. Although avocados are technically fruits, the United States Department of Agriculture classifies them as vegetables based on their nutrient composition and culinary use. Higher fruit and vegetable intake during pregnancy was associated with a lower risk of GDM [3]. Interestingly, Sun et al. found that consuming a specific fruit (i.e., grape) but not total fruit intake during pregnancy was associated with lower GDM risk [4]. In contrast, evidence regarding hypertensive disorders, such as preeclampsia and gestational hypertension, is more consistent. A meta‐analysis of 12 studies found that adherence to a healthy dietary pattern was associated with significantly lower odds of hypertensive disorders of pregnancy, while unhealthy dietary patterns were associated with increased risk [1]. These findings were echoed by Kibret et al., who also found a protective association between healthy diets and hypertensive disorders in pregnancy [2]. The absence of significant findings in this study may, in part, be due to the relatively low level of avocado intake among participants. The average intake among consumers was 19.5 g per day—approximately one‐eighth of a medium avocado. It is possible that this amount was insufficient to produce measurable effects on other pregnancy outcomes. Future studies should consider whether higher or more frequent avocado consumption may be required to observe broader clinical benefits during pregnancy.

Similarly, no significant associations were found between avocado consumption during pregnancy and newborn outcomes. Similar to maternal outcomes, there is no existing literature specifically linking avocado intake to newborn outcomes. However, the influence of broader dietary patterns—including fruit and vegetable consumption—on neonatal outcomes has been well studied. Generally, higher adherence to a healthy diet during pregnancy is associated with more favorable newborn outcomes, though findings are sometimes mixed. A meta‐analysis by Abdollahi et al. found that mothers adhering to a healthy diet had infants with higher birth weights and lower odds of low birth weight and preterm birth, but no significant associations were observed for LGA, SGA, or macrosomia [1]. Similarly, another meta‐analysis reported that healthy dietary patterns were associated with a reduced risk of preterm birth and a trend toward lower SGA risk, but not with birth weight [65]. Kibret et al. also reported consistent findings for preterm birth [2]. Evidence from a Danish cohort showed that both fruit and vegetable intake during pregnancy were positively associated with birth weight [66]. However, the Infancia y Medio Ambiente cohort found an inverse association between vegetable intake—but not fruit intake—and SGA [67], while Martínez‐Galiano et al. reported the opposite: a significant association between fruit intake—but not vegetable intake—and reduced risk of SGA [68]. Except for melon, individual fruits (e.g., orange, banana, apple, strawberry, cherry, peach, watermelon, kiwi fruit, and grapes) and vegetables (e.g., swiss chard, cabbage, lettuce, tomato, carrot, green bean, eggplants, pepper, asparagus, cold vegetable soup, onion, garlic, and mushroom) were not significantly associated with SGA [68]. The lack of significant findings in this study may be explained by low avocado consumption among consumers. For instance, Martínez‐Galiano et al. found that only women in the highest quintile of fruit intake (> 420 g/day) had a significantly lower risk of SGA compared to those in the lowest quintile (≤ 121 g/day) [68]. It is possible that a certain threshold of produce intake is necessary to observe meaningful effects on newborn outcomes.

Strengths of this study include its prospective design, detailed dietary assessment using a validated FFQ, adjustment for potential confounding variables, the relatively large sample size, and registry‐based outcome data. Nonetheless, some limitations should be acknowledged. First, FFQ relies on memory and may be prone to recall bias. In addition, due to the observational nature of the study, causality cannot be inferred, and residual confounding may persist. Although sensitivity analyses using stepwise adjustment, IPTW weighting, and E‐value assessment produced consistent results, suggesting robustness to measured covariates, avocado consumption may still be a marker of broader health‐conscious behaviors that were not fully captured by the included covariates. Moreover, the association was observed for cesarean delivery but not for other pregnancy outcomes, such as gestational diabetes or hypertensive disorders. If avocado intake had a generalized effect on maternal metabolic health, associations with these outcomes might also have been expected. The absence of such associations weakens support for a specific biological effect of avocado intake. Therefore, residual confounding and healthy‐user bias remain possible explanations for these findings. Second, given the number of pregnancy and newborn outcomes examined, the potential for a Type I error due to multiple comparisons cannot be excluded. Notably, the observed association between avocado consumption and cesarean delivery would not remain statistically significant after applying a conservative Bonferroni correction. Therefore, this finding should be interpreted as exploratory and warrants confirmation in independent populations. Third, although avocado intake was primarily modeled as a binary variable due to its episodic consumption, additional analyses using categorized intake groups and continuous intake were conducted. These analyses did not demonstrate a clear dose–response pattern: moderate intake (> 0–8 g/day) was associated with lower odds of cesarean delivery, whereas higher intake (> 8 g/day) was not, and no significant association was observed when avocado intake was modeled continuously. This non‐monotonic pattern may reflect the highly skewed distribution of avocado intake and limited statistical precision from the relatively small number of cesarean delivery events; however, Type I error, residual confounding, and dietary measurement error may also have contributed to this pattern. Furthermore, the average avocado intake among consumers was relatively modest (19.5 g/day), and it remains uncertain whether this amount would be sufficient to meaningfully influence biological pathways related to labor and delivery. The absence of a clear dose–response relationship and the relatively modest intake levels limit inference regarding the magnitude and biological plausibility of the observed association. Further research is needed to determine whether this association is reproducible and biologically plausible. Fourth, since the OGTT was not administered to all pregnant women, it is possible that some pregnant women were misclassified as not having GDM. However, the proportion of women with GDM was slightly higher than the 2019 Finnish national estimate as highlighted above. In addition, this study included only pregnant women anticipated to deliver at KUH and excluded participants due to unreliable FFQ data. However, prior evaluations of the KuBiCo cohort have demonstrated that participants are broadly representative of the general Finnish pregnant population with respect to key maternal and birth characteristics, supporting the generalizability of the findings [22]. Fifth, although cesarean section subtype (elective, urgent, and emergency) was available in the dataset, the small number of cases limited the ability to examine the associations by cesarean section type. Finally, while some authors are affiliated with the Avocado Nutrition Center, steps were taken to ensure transparency and analytical independence, including preregistration of the study protocol, independent data analysis conducted by investigators based in Finland, and adherence to established reporting guidelines (STROBE) to ensure transparent reporting.

Maternal avocado consumption during pregnancy was associated with lower odds of cesarean delivery, independent of maternal and lifestyle factors, while no significant associations were observed for other pregnancy or newborn outcomes. This association should be interpreted within the context of the study design. The findings suggest a potential relationship between avocado intake and mode of delivery; however, the underlying mechanisms remain unclear. Although sensitivity analyses yielded consistent results, the findings may still be influenced by residual confounding and potential selection bias. Further research is needed to confirm these findings and to better understand potential dose–response relationships and underlying mechanisms.

## Author Contributions

**Feon W. Cheng:** conceptualization, methodology, data curation, investigation, validation, supervision, visualization, project administration, resources, writing – original draft, writing – review and editing. **Nikki A. Ford:** conceptualization, methodology, supervision, project administration, writing – review and editing. **Katri Backman:** conceptualization, methodology, investigation, writing – review and editing. **Raimo Voutilainen:** conceptualization, methodology, investigation, writing – review and editing. **Markku Pasanen:** conceptualization, methodology, investigation, writing – review and editing. **Leea Keski‐Nisula:** conceptualization, methodology, investigation, funding acquisition, writing – review and editing. **Soili M. Lehto:** conceptualization, methodology, investigation, writing – review and editing. **Sari Hantunen:** conceptualization, methodology, software, data curation, investigation, validation, formal analysis, writing – original draft, writing – review and editing.

## Funding

The KuBiCo Study has been funded by the Research Committee of the Kuopio University Hospital Catchment Area for the State Research Funding and The Foundation for Pediatric Research.

## Ethics Statement

The Research Ethics Committee of The Wellbeing Services County of Central Finland has reviewed and approved the KuBiCo protocol (dated 8 December 2011, Dnro 18U/2011).

## Consent

All KuBiCo participants provided their written informed consent to participate in this study.

## Conflicts of Interest

N.A.F. and F.W.C. are employees of the Avocado Nutrition Center. The remaining authors declare no conflicts of interest.

## Supporting information


**Data S1:** STROBE Statement—checklist of items that should be included in reports of observational studies.

## Data Availability

The data supporting the results of this study include pseudonymized patient‐level clinical data obtained from research questionnaires and hospital patient records. Therefore, the data cannot be made publicly available. All raw data from questionnaires and biological samples are collected and stored in the electronic KuBiCo database (eKuTKa), from which research partners and international collaborators can obtain data for research use after mutual agreements. The KuBiCo Advisory Board grants research groups permissions to use KuBiCo data. Requests for use of pseudonymized data may be considered on a case‐by‐case basis and require approval by the institutional ethics committee and compliance with data protection regulations.
